# The Synchrony-Prosociality Link Cannot Be Explained Away as Expectancy Effect: Response to Atwood et al. (2022)

**DOI:** 10.1162/opmi_a_00103

**Published:** 2023-09-20

**Authors:** Bahar Tunçgenç, Joshua S. Bamford, Christine Fawcett, Emma Cohen

**Affiliations:** Psychology Department, Nottingham Trent University, Nottingham, UK; Social Body Lab, Institute of Human Sciences, University of Oxford, Oxford, UK; Centre of Excellence in Music, Mind, Body and Brain, University of Jyväskylä, Jyväskylä, Finland; Department of Psychology, Stockholm University, Stockholm, Sweden; Wadham College, Oxford, UK

**Keywords:** synchrony, prosociality, expectancy, experimenter bias, placebo effects, development, social interactions

## Abstract

Moving in time to others, as is often observed in dance, music, sports and much of children’s play cross-culturally, is thought to make people feel and act more prosocially towards each other. In a recent paper, Atwood et al. (2022) argued that the inferential validity of this link found between synchronous behaviour and prosociality might be mainly due to “expectancy effects generated by a combination of (1) experimenter expectancy, leading to experimenter bias; and (2) participant expectancy (i.e., placebo effects)”. Here, we counter these arguments with (1) examples of studies devoid of experimenter expectancy effects that nevertheless demonstrate a positive link between synchrony and prosociality, and (2) insights from the developmental literature that address participant expectancy by showing how expectations formed through lived experiences of synchronous interactions do not necessarily threaten inferential validity. In conclusion, there is already sufficient good-quality evidence showing the positive effects of synchronous behaviours on prosociality beyond what can be explained by experimenter or participant expectation effects.

In their recent paper, Atwood et al. (2022) call into question evidence for a causal association between behavioural synchrony (such as in dance and music-making) and prosociality. In a critical review of the literature, they suggest that synchrony and prosociality are linked by nothing more than mere expectancy, specifically “expectancy effects generated by a combination of (1) experimenter expectancy, leading to experimenter bias; and (2) participant expectancy (i.e., placebo effects)” (Atwood et al., 2022). They also report a novel study with results apparently supporting their second conjecture. While acknowledging the possibility for experimenter bias and expectancy effects in synchrony-prosociality research, we argue that a more comprehensive review of the relevant literature renders this interpretation unlikely. In this brief response, we counter Atwood et al.’s arguments: (1) with examples of well-designed studies addressing experimenter expectancy effects that were given short shrift or omitted from Atwood et al.’s critical review, and (2) by referencing the developmental literature, which reveals synchrony-prosociality effects that cannot obviously be explained in terms of participant expectancy. While more studies are needed to identify what causal pathways link behavioural synchrony and prosociality, we believe that there is already sufficient good-quality evidence to suggest that acting together in time produces prosocial effects beyond those owing to experimenter bias or prior participant expectations that synchrony is associated with prosocial effects.

Atwood et al.’s first critique about experimenter expectancy effects can be countered by compelling evidence that the synchrony-prosociality link is observed in conditions that carefully control for experimenter bias. The review and meta-analysis by Rennung and Göritz (2016), on which Atwood et al. (2022) rely heavily, found that while potential for experimenter bias nullified the effect of synchrony on prosocial *behaviour*, there was still a positive effect of synchrony on prosocial *attitudes* even when experimenter bias was controlled for (i.e., when the experimenter was unaware of the hypotheses/condition). Since then, many more examples of experiments that mitigate for experimenter bias have been published. For example, Cirelli et al. (2016) observed positive effects of synchrony on prosocial behaviour in 14-month-old infants following synchronous vs non-synchronous bouncing. In this design, experimenter bias was mitigated by ensuring that different experimenters were present during the synchronous movement task and the prosociality task. Still, positive effects of synchrony on prosocial behaviour were observed. In addition, studies with infants using puppets to manipulate synchronous movement show similar effects without any possibility of experimenter facial expressions or other bias cuing infants to the positivity of synchronous movement (Fawcett & Tunçgenç, 2017; Tunçgenç et al., 2015). Virtual Reality technology has also enabled greater experimental control in interpersonal synchrony studies. Participants in Tarr et al. (2018) immersive Virtual Reality study performed movements that were either matched in time or not by avatars that appeared to be in the same room, in a between-participants, double-blind experimental design. Participants whose movements were matched in time reported increased social closeness with their synchronous virtual partners, compared with participants whose partners’ movements appeared to be non-synchronous (matched in form but not in time). In addition to this well-established social closeness measure, some novel measures were included in this study. In contrast to the primary measure of social closeness, effects of the manipulation on these novel measures were non-significant. These novel measures lacked precedent in studies of interpersonal synchrony (see Vicaria & Dickens, 2016, for review), and may be unreliable in studies of this type. For instance, Bamford et al. (2023) similarly found no effect of interpersonal distance, while self-report and social attention measures were significant, which could be due to participants feeling constrained in their use of space in a lab-based task. Atwood et al. reference Tarr et al. (2018) as an example of a study that took steps to decrease bias but go on to dismiss its results as “mixed”, despite the primary measure showing a clear effect. While this interpretation of “mixed” findings suits their argument that the synchrony-prosociality effect relies on expectancy effects, we contend that this argument does not rely on a balanced review of the available evidence. Overall, extant literature shows that the synchrony – prosociality link is maintained even after controlling for experimenter bias, which could inflate the true effect of synchrony.

Atwood et al.’s second critique attributes the apparent synchrony-prosociality link to culturally received belief, rather than to any integral causal mechanism (e.g., self-other overlap …). That is, they suggest that the effect simply comes down to participants’ expectations that synchrony will make them more prosocial and that these expectations are acquired via received cultural wisdom. A wealth of developmental psychology literature (largely overlooked in Atwood et al.’s paper) suggests that this is not the case. This literature provides compelling evidence that the origins of the association between synchrony and prosociality are rooted in infants’ early social interactions. Infants spend about 65% of their time actively interacting with others (Užgiris et al., 1989). Often, these people are caregivers who are closely bonded with the infant. During their social interactions already within the first year of life, infants maintain eye contact, follow their interlocutor’s head direction and eye gaze, share attention, take turns, coordinate movements, and become more responsive to others’ prompts and needs (Tomasello et al., 2005). In turn, caregivers frequently imitate and align the timing of their movements to the infant, which results in highly coordinated, synchronous exchanges (de Barbaro et al., 2013; de Klerk et al., 2019; Ray & Heyes, 2011), even if not necessarily rhythmical or time-locked in nature. It is through these reciprocal, synchronous exchanges that infants’ nervous systems and social-cognitive skills develop further; they learn to better coordinate their movements with others, understand others’ emotional and mental states, regulate their own internal states, and establish inter-subjectivity (Feldman, 2007; Redcay & Warnell, 2018; Tunçgenç, 2017). Longitudinal studies show that experiencing interactional synchrony with caregivers in infancy is related to better cognitive and communicative skills, social-emotional adaptation (Jaffe et al., 2001), and more secure attachment (Isabella et al., 1989) later in childhood. While we have yet to understand precisely how coordination in infancy is linked with rhythmic synchronisation abilities in childhood, the existing developmental literature reveals a picture showing that infants and children come to associate synchrony with positive social engagement and affiliation from the bottom-up – through everyday experiences of social interactions, which frequently feature highly coordinated, synchronous movements.

To demonstrate the potential for participant expectancy effects, Atwood et al. asked participants to imagine a hypothetical scenario in which a group of college students in a psychology experiment walk in step together around a college campus, or just walk together around a college campus. Then, participants responded to a series of questions about how those people might feel in the different conditions. Participants judged that the hypothetical walkers would feel more connected, trusting, similar, on the same team, coordinated and synchronised after walking in step with one another, as compared to the control condition, supposedly supporting the authors’ claims. It is already known, however, that imagining a behaviour may yield similar effects to directly experiencing it due to shared neurocognitive mechanisms (Rizzolatti & Craighero, 2004). Moreover, the participants in Atwood et al.’s study were university students who may have been familiar with common experimental paradigms in social psychology, potentially leading to responses based on demand characteristics (i.e., participants responding in a way that they believe the experimenter wants them to answer). To check this, cover stories for the study, filler questions unrelated to the outcome variables, and a hypothesis probe could be used, none of which were included in Atwood et al.’s study.

Previous research has shown that infants, who are highly unlikely to be affected by such demand characteristics, expect that people who move in synchrony will be socially closer to each other from 15 months of age (Fawcett & Tunçgenç, 2017) and are inclined to affiliate with synchronous others by 12 months of age (Tunçgenç et al., 2015). Crucially, infants did not show the same reactions when they were 3 months younger (12 and 9 months, respectively), indicating the role of learning these expectations via shared synchronous experiences. Thus, far from “mistaken beliefs about synchrony’s impact on prosociality”, we argue, based on the developmental science reviewed above, that these associations track the real thing – that synchrony has a causal role in affiliative sociality and even babies recognise that. If so, participant expectancy is not an inherent threat to inferential validity; it is only when participant expectancy leads to demand characteristics that it becomes problematic.

In conclusion, experimenter bias and participant expectancy do not explain away the synchrony-prosociality effect. Studies that have eliminated experimenter bias continue to find a positive impact of synchronous movement on prosociality. The expectation that synchrony and prosociality are linked is not a result of spurious received wisdom. Instead, people learn via social interactions throughout their lifespan that increased prosociality and bonding tend to follow from reciprocal, synchronous interactions – just as they might expect to feel more bonded after laughing, crying, embracing or enduring agonising pain together. In other words, people expect things that happen to happen. In the case of the synchrony – prosociality link, it is possible that top-down expectations may play some role in the observed effects of synchrony on prosociality, and we agree that research designs need to account for this possibility. However, to claim that expectations are “substantial” or are the more interesting part of the story is to ignore the literature on the origins of these expectations. In light of these insights, Atwood et al.’s (2022) findings are more parsimoniously explained as people’s expectations meeting the reality.

## References

[bib1] Atwood, S., Schachner, A., & Mehr, S. A. (2022). Expectancy effects threaten the inferential validity of synchrony-prosociality research. Open Mind, 6, 280–290. 10.1162/opmi_a_00067, 36891035 PMC9987344

[bib2] Bamford, J. S., Burger, B., & Toiviainen, P. (2023). Turning heads on the dance floor: Synchrony and social interaction using a silent disco paradigm. Music and Science, 6, 1–13. 10.1177/20592043231155416

[bib3] Cirelli, L. K., Wan, S. J., & Trainor, L. J. (2016). Social effects of movement synchrony: Increased infant helpfulness only transfers to affiliates of synchronously moving partners. Infancy, 21(6), 807–821. 10.1111/infa.12140

[bib4] de Barbaro, K., Johnson, C. M., & Deák, G. O. (2013). Twelve-month social revolution emerges from mother-infant sensorimotor coordination: A longitudinal investigation. Human Development, 56(4), 223–248. 10.1159/000351313

[bib5] de Klerk, C., Lamy-Yang, I., & Southgate, V. (2019). The role of sensorimotor experience in the development of mimicry in infancy. Developmental Science, 22(3), Article e12771. 10.1111/desc.12771, 30415485 PMC6767077

[bib6] Fawcett, C., & Tunçgenç, B. (2017). Infants’ use of movement synchrony to infer social affiliation in others. Journal of Experimental Child Psychology, 160, 127–136. 10.1016/j.jecp.2017.03.014, 28427721

[bib7] Feldman, R. (2007). Parent–infant synchrony: Biological foundations and developmental outcomes. Current Directions in Psychological Science, 16(6), 340–345. 10.1111/j.1467-8721.2007.00532.x

[bib8] Isabella, R. A., Belsky, J., & von Eye, A. (1989). Origins of infant-mother attachment: An examination of interactional synchrony during the infant’s first year. Developmental Psychology, 25(1), 12–21. 10.1037/0012-1649.25.1.12

[bib9] Jaffe, J., Beebe, B., Feldstein, S., Crown, C. L., & Jasnow, Michael D. (2001). Rhythms of diologue in infancy: Coordinated timing in development. Monographs of the Society for Research in Child Development, 66(2), i–viii. 11428150

[bib10] Ray, E., & Heyes, C. (2011). Imitation in infancy: The wealth of the stimulus. Developmental Science, 14(1), 92–105. 10.1111/j.1467-7687.2010.00961.x, 21159091

[bib11] Redcay, E., & Warnell, K. R. (2018). A social-interactive neuroscience approach to understanding the developing brain. Advances in Child Development and Behavior, 54, 1–44. 10.1016/bs.acdb.2017.10.001, 29455860

[bib12] Rennung, M., & Göritz, A. S. (2016). Prosocial consequences of interpersonal synchrony. Zeitschrift für Psychologie, 224(3), 168–189. 10.1027/2151-2604/a000252, 28105388 PMC5137339

[bib13] Rizzolatti, G., & Craighero, L. (2004). The mirror-neuron system. Annual Review of Neuroscience, 27, 169–192. 10.1146/annurev.neuro.27.070203.144230, 15217330

[bib14] Tarr, B., Slater, M., & Cohen, E. (2018). Synchrony and social connection in immersive Virtual Reality. Scientific Reports, 8(1), Article 3693. 10.1038/s41598-018-21765-4, 29487405 PMC5829252

[bib15] Tomasello, M., Carpenter, M., Call, J., Behne, T., & Moll, H. (2005). Understanding and sharing intentions: The origins of cultural cognition. The Behavioral and Brain Sciences, 28(5), 675–691. 10.1017/S0140525X05000129, 16262930

[bib16] Tunçgenç, B. (2017). Movement synchrony, joint actions, and collective agency in infancy. In N. J. Enfield & P. Kockelman (Eds.), Distributed agency (pp. 169–178). Oxford University Press. 10.1093/acprof:oso/9780190457204.003.0018

[bib17] Tunçgenç, B., Cohen, E., & Fawcett, C. (2015). Rock with me: The role of movement synchrony in infants’ social and nonsocial choices. Child Development, 86(3), 976–984. 10.1111/cdev.12354, 25702860

[bib18] Užgiris, I. Č., Benson, J. B., Kruper, J. C., & Vasek, M. E. (1989). Contextual influences on imitative interactions between mothers and infants. In Action in social context: Perspectives on early development (pp. 103–128). Springer. 10.1007/978-1-4757-9000-9_4

[bib19] Vicaria, I. M., & Dickens, L. (2016). Meta-analyses of the intra- and interpersonal outcomes of interpersonal coordination. Journal of Nonverbal Behavior, 40(4), 335–361. 10.1007/s10919-016-0238-8

